# Suppression of the *HOS1* Gene Affects the Level of ROS Depending on Light and Cold

**DOI:** 10.3390/life13020524

**Published:** 2023-02-14

**Authors:** Tatiana Y. Gorpenchenko, Galina N. Veremeichik, Yurii N. Shkryl, Yulia A. Yugay, Valeria P. Grigorchuk, Dmitry V. Bulgakov, Tatiana V. Rusapetova, Yulia V. Vereshchagina, Anastasiya A. Mironova, Evgeniyy P. Subbotin, Yuriy N. Kulchin, Victor P. Bulgakov

**Affiliations:** 1 Federal Scientific Center of the East Asia Terrestrial Biodiversity, Far Eastern Branch of the Russian Academy of Sciences, 159 Stoletija Str., 690022 Vladivostok, Russia; 2 Institute of Automation and Control Processes, Far Eastern Branch of the Russian Academy of Sciences, 5 Radio Str., 690041 Vladivostok, Russia

**Keywords:** Arabidopsis, HOS1, cold stress, high light stress, reactive oxygen species, intracellular ROS accumulations, RbohD, RbohF, Apx1, Apx2

## Abstract

The E3 ubiquitin-protein ligase HOS1 is an important integrator of temperature information and developmental processes. HOS1 is a negative regulator of plant cold tolerance, and silencing *HOS1* leads to increased cold tolerance. In the present work, we studied ROS levels in *hos1*^Cas9^ *Arabidopsis thaliana* plants, in which the *HOS1* gene was silenced by disruption of the open reading frame via CRISPR/Cas9 technology. Confocal imaging of intracellular reactive oxygen species (ROS) showed that the *hos1* mutation moderately increased levels of ROS under both low and high light (HL) conditions, but wild-type (WT) and *hos1*^Cas9^ plants exhibited similar ROS levels in the dark. Visualization of single cells did not reveal differences in the intracellular distribution of ROS between WT and *hos1*^Cas9^ plants. The *hos1*^Cas9^ plants contained a high basal level of ascorbic acid, maintained a normal balance between reduced and oxidized glutathione (GSH and GSSG), and generated a strong antioxidant defense response against paraquat under HL conditions. Under cold exposure, the *hos1* mutation decreased the ROS level and substantially increased the expression of the ascorbate peroxidase genes *Apx1* and *Apx2*. When plants were pre-exposed to cold and further exposed to HL, the expression of the NADPH oxidase genes *RbohD* and *RbohF* was increased in the *hos1*^Cas9^ plants but not in WT plants. *hos1*-mediated changes in the level of ROS are cold-dependent and cold-independent, which implies different levels of regulation. Our data indicate that *HOS1* is required to maintain ROS homeostasis not only under cold conditions, but also under conditions of both low and high light intensity. It is likely that *HOS1* prevents the overinduction of defense mechanisms to balance growth.

## 1. Introduction

The pleiotropic regulator HOS1 (E3 ubiquitin-protein ligase; synonym: high expression of osmotically responsive genes 1) is currently attracting attention from researchers as a link between the developmental processes of plants and abiotic stresses. This protein is considered a key integrator of temperature information and developmental processes that are essential for plant survival [1]. HOS1 is necessary to adapt the development of plants both to short-term cold stress and freezing tolerance by interacting with transcription factor ICE1 (Inducer of CBF expression 1), an important factor in establishing cold resistance [2,3,4,5]. ICE1 induces genes encoding dehydration-responsive element-binding proteins (CBFs), such as CBF1, CBF2, and CBF3, by binding to gene promoters [6,7]. This leads to the acquisition of resistance to cold through increased expression of numerous cold-responsive (*COR*) genes. In *hos1* mutants, ICE1 is not degraded [3], which leads to the acquisition of cold tolerance via the unified ICE-CBF pathway [6,7]. If HOS1 is a negative regulator of cold stress, acting mainly by inhibiting ICE1 [3,4], then it is a positive regulator of heat stress [8]. In *Arabidopsis*, the *hos1* mutation not only increased cold tolerance, but also induced flavonoid biosynthesis [9].

HOS1 is also involved in light signaling [10,11,12]. HOS1 physically interacts with the zinc-finger protein CONSTANS (CO) to regulate flowering time [10]. To link cold and photoperiodic responses, HOS1 interacts simultaneously with phytochrome B (phyB) and CO [11]. phyB activates HOS1, and HOS1 subsequently inhibits the transcriptional activity of the phytochrome-interacting transcription factor PIF4 [12]. *hos1* mutants demonstrated an early flowering phenotype [10] and exhibited elongated hypocotyls [12].

Light plays a significant role in the adaptation of plants to abiotic stresses, and cold and light signaling combine to optimize plant growth and development [13,14,15,16,17]. The balance between ROS production and ROS decomposition, mediated by ROS-scavenging systems, determines the acclimation state. Signaling ROS are generated in a response to stress by NADPH oxidases (Rboh) at the plasma membrane and activate an acclimation response [18]. Besides acclimation, ROS are necessary for rapid systemic signaling. The balance between ROS production and ROS decomposition, mediated by the ROS-scavenging systems, determines the acclimation state. Cold acclimation increases plant tolerance to excessive light by increasing photosynthetic capacity during high light treatment [19,20]. Signaling modules linking cold adaptation to intense light have been summarized by Roeber et al. [17]. phyB plays a role in these links because it perceives both light and ambient temperature signals [15,17]. Cold acclimation transcription factors, CBFs, interact with PIF3 under cold stress, thereby preventing phyB degradation. Cold-stabilized phyB acts downstream of CBFs to positively regulate freezing tolerance by modulating the expression of stress-responsive genes. Devireddy et al. [21] showed that phyB is required for ROS wave initiation after light stress, and phyB acts via *Arabidopsis* RbohD activation. *RbohD* and *RbohF* are both required for local and systemic ROS signaling during light stress [15,17,21].

Although the combination of heat and intense light is more common in plant cultivation, the combination of cold and intense light is also important [22]. We proceeded from the assumption that *HOS1* may be involved in the regulation of ROS metabolism, since ROS play an important role in both light and temperature adaptation [13,14,15,16,17]. Therefore, the purpose of this work was to study ROS production in WT and *hos1*^Cas9^ mutant *Arabidopsis* plants.

Recently, CRISPR/Cas9-engineered *Arabidopsis* plants were established, in which frameshift indels were introduced in the first exon of *HOS1* [9]. This resulted in the appearance of premature stop codons, which completely disrupted the open reading frame of the gene [9]. These plants, designated as *hos1*^Cas9^ plants, were compared with SALK T-DNA insertion mutant plants, line *hos1-3* [10]. The *hos1-3* mutation is located in the fifth exon and disrupts the *HOS1* reading frame at position 912 bp of mRNA, leading to the production of a truncated protein that retains 304 aa of its native form. The *hos1*^Cas9^ and *hos1-3* mutations similarly increased cold resistance and *CBF* gene expression, but their effect on secondary metabolism was different [9]. The *hos1-3* mutant line was also used in the present work to compare the results.

In the present investigation, *hos1*^Cas9^ plants were grown under normal and high light (HL) conditions, with or without cold pre-treatment. *hos1*^Cas9^ plants demonstrated different ROS levels compared with WT plants under these treatments. The *hos1* mutation caused moderate ROS elevation at normal growth temperatures. In conditions of cold treatment followed by HL treatment, ROS levels decreased, and this process was accompanied by an increase in *RbohD* and *RbohF* expression with a concomitant increase in the activity of antioxidant genes.

## 2. Materials and Methods

### 2.1. Plant Material

The seeds of the *A. thaliana* wild-type (WT) Col-0 ecotype were purchased from the RIKEN BioResource Research Center (Ibaraki, Japan), while the *hos1*^Cas9^ mutant lines were obtained and described by us earlier [9]. In the present study, the *hos1-1*^Cas9^ line was used. Surface-sterilized seeds were subjected to cold stratification for 1 day at 4 °C prior to planting on agar plates with half-strength Murashige and Skoog medium and grown under long-day conditions (16 h light/8 h dark) in a growth chamber at 25 °C. After 2 weeks, seedlings were planted in the soil to promote rosette production and flowering. Four-week-old plants were used for experiments. *HOS1* was disabled by genome editing, resulting in the termination of HOS1 translation, which was confirmed by heteroduplex mobility assay (HMA), high-resolution melting analysis (HRM) and genomic DNA sequencing of stable *Arabidopsis hos1*^Cas9^ mutants [9]. The first exon contained a premature stop codon, which completely disrupted the open reading frame of the *HOS1* gene [9]. Silencing of the HOS1 protein was also monitored by Western blot before starting the experiments (Supplementary Figure S1). *A*. *thaliana* SALK_069312C T-DNA insertion line *hos1-3* [10] was purchased from the Salk Institute for Biological Studies, San Diego, CA, USA. The *hos1-3* line was cultivated using the same conditions.

The callus lines were obtained from leaves of WT and *hos1*^cas9^ mutant plants. The control and *hos1*^cas9^ calli were cultivated as described [23] using W_2,4-D_ medium supplemented with 0.4 mg/L 2,4-dichlorophenoxyacetic acid in the dark at 24 °C with 25-day subculture intervals. The W_2,4-D_ medium contained standard Murashige and Skoog macrosalts, microsalts, and Fe-EDTA, with the exception of NH_4_NO_3_, the concentration of which was decreased up to 400 mg/L. The following components were added to the W_2,4-D_ medium (mg/L): thiamine-HCl (0.2), nicotinic acid (0.5), pyridoxine-HCl (0.5), meso-inositol (100), peptone (100), sucrose (25000), and agar (6000). All reagents were obtained from Sigma-Aldrich (St. Louis, MO, USA, “Tissue Culture Grade”). Transgenic calli were termed according to the number of the original *hos1* plants (*hos1-1*^Cas9^ line) from which they were originated.

### 2.2. Experimental Design for High Light Stress Treatment

The experiments were carried out in a specially designed light unit, which was placed in a growth chamber. We used warm white LED lamps that were located at the top of the chamber, and the cool air came from the sides. Based on the literature data, the density of the photosynthetic photon flux (PPFD) was selected at a level of 80 µmol m^−2^ s^−1^ as control conditions, and 1200 µmol m^−2^ s^−1^ in the experiments for intense illumination, designated here as “high light” (HL) conditions. Plants were exposed to high light treatments for 15, 30, and 60 min, as well as for 2 h. Spectral characteristics of the light-emitting diode lamps used in experiments are shown in Supplementary Figure S2. Cold treatment (12 °C) was performed for 24 h under normal light and HL (Supplementary Table S1).

### 2.3. Laser Confocal Imaging of Intracellular ROS

Measurements of intracellular ROS were performed as previously described [24]. The experiments were based on the ability of plant cells to oxidize fluorogenic dyes to their corresponding fluorescent analogues, allowing for ROS determination in living cells. Epidermal cells from the abaxial leaf side were analyzed. The middle part of the leaves, after excision, were cut and incubated in liquid MS/2 medium containing 50 µM 2,7-dichlorodihydrofluorescein diacetate (H_2_DCF-DA, Molecular Probes, Eugene, OR, USA) or 10 µM dihydrorhodamine 123 (H2R123; Molecular Probes, Eugene, OR, USA) at 24 ± 1 °C in the dark for 10 min. Subsequently, parts of the leaves were washed twice and placed in the MS/2 medium in a POC chamber (a convenient live cell incubation device). Examination of DCF fluorescence in single living cells was performed with the LSM 510 META confocal laser scanning microscope (Carl Zeiss, Germany) equipped with an argon laser with an effective power of 30 mW and intensity of 3%. DCF fluorescence was measured at an excitation wavelength of 488 nm and detected with a bandpass emission filter at 505–530 nm. Confocal images were recorded in a 40 s time series at intervals of 0.5 ms. The autofluorescence of chloroplasts was recorded with an additional emission channel at 600 nm. The objective was a Plan-Neofluar 40 × 1.3. The maximum intensity projection mode was applied to obtain 2D images from the time series. Video files of the captured images were recorded and analyzed using LSM 510 Release 3.5 software (Carl Zeiss, Germany) and for automatic whole image calculation ZEN2012 software (Blur edition) (Carl Zeiss, Göttingen, Germany). Data were presented as the average of DCF fluorescence intensity and pixels from three separate experiments (30–40 cells were analyzed in each experiment).

### 2.4. Paraquat and Argon Laser Treatments

*Arabidopsis* plants were grown for 28 days in control conditions, and then were treated with paraquat (15 μM, final concentration) for 1 h under control conditions or HL. Light stress was caused by continuous illumination of cells with the LSM 510 META Ar laser (excitation at 488 nm) as described [24]. The effective power was 30 mW. The laser intensity was increased by 1.5 times compared to the standard intensity.

### 2.5. Measurement of Glutathione and Ascorbic Acid

#### 2.5.1. Chemicals

Analytical standards: ascorbic acid (AsA) was obtained from Sigma-Aldrich (St. Louis, MO, USA); reduced glutathione (GSH) and oxidized glutathione (GSSG) were obtained from BioChemica (PanReac AppliChem, Ottoweg, Germany). All standard solutions, extraction buffers, and eluents were prepared with Milli-Q water (Millipore, Bedford, MA, USA). HPLC-grade acetonitrile was obtained from PanReac AppliChem (Ottoweg, Germany). All other chemicals used were of analytical grade.

#### 2.5.2. Sample Preparation for AsA and Glutathione Assays

For sample preparation, 35-day-old *Arabidopsis* plants were selected for analysis for AsA and glutathione after light treatment. Frozen plant tissues were ground with a mortar and pestle in liquid nitrogen and immediately homogenized with two volumes (*w*/*v*) of cold (4 °C) extraction solution (2% trifluoroacetic acid). Then, the homogenates were sonicated on ice and centrifuged at 15,000× *g* for 20 min at 4 °C. The supernatants were collected, filtered through a 0.45 μm nylon membrane (Millipore, Bedford, MA, USA), and immediately analyzed by LC-UV-MS/MS. All manipulations were carried out in a cold room at 4 °C and protected from light. Each sample was prepared in two replications.

#### 2.5.3. Analytical Chromatography and Mass Spectrometry

The LC-UV-MS/MS assays were carried out at the Instrumental Centre of Biotechnology and Gene Engineering of Federal Scientific Center of East Asia Terrestrial Biodiversity using a 1260 Infinity analytical HPLC system (Agilent Technologies, Santa Clara, CA, USA). The separation was performed using an analytical Zorbax C18 column (150 mm, 2.1 mm i.d., 3.5 μm part size, Agilent Technologies, Santa Clara, CA, USA) in isocratic conditions with a mixture of acetonitrile:water (3:97) with formic acid (0.1%) at a flow rate of 0.2 mL/min. The column temperature was maintained at 35 °C. UV spectra were obtained with a DAD detector in the range between 200 and 400 nm. Chromatographic data for AsA quantification were recorded at a wavelength of 243 nm. Instrument operation, data collection, and analysis were controlled using the Agilent OpenLAB CDS software (v.01.06.111).

The LC system was interfaced with a mass spectrometer, the Bruker HCT ultra PTM Discovery System (Bruker Daltonik GmbH, Bremen, Germany), to detect glutathiones in plant tissue samples. The HCT ultra is equipped with a high-capacity ion trap that enables the acquisition of tandem mass spectrometry data of low-abundance precursor ions. The analyses were carried out using electrospray ionization in the mass range ultrascan mode with positive ionic polarity and a detection range of *m/z* 100–400. The following instrument parameters were applied: the drying gas (N_2_) flow rate was 8.0 L/min, the nebulizer gas (N_2_) pressure was 25 psi, the ion source potential was −4.0 kV, and the drying gas temperature was 325 °C. Manual MS/MS acquisition was chosen for quantification of the glutathiones using the following *m/z* values of isolation masses: 308 for GSH determination and 613 for GSSG. MS data were collected using Bruker Daltonics Compass 1.3 esquire (v.6.2.581.3) management software and processed with Bruker Daltonics Compass 1.3 (v.4.0.234.0) data analysis software.

Quantification was performed by obtaining calibration curves with linear regression (correlation coefficient R^2^ above 0.997) built at five concentration levels with standards of AsA, GSH, and GSSG. Standard solutions were prepared in 2% trifluoroacetic acid under cold, dark conditions at 4 °C. The following concentration ranges were chosen for calibration: 520–0.1 nmol/mL for GSH, 110–0.1 nmol/mL for GSSG, and 5000–1 nmol/mL for AsA. The calibration was confirmed several times during the analysis.

### 2.6. RNA Isolation, cDNA Synthesis, and PCR Reactions

To isolate total RNA, a LiCl precipitation protocol was used [23]. The integrity and purity of RNA samples were tested using microcapillary electrophoresis chips (Experion, Bio-Rad Laboratories Inc., Hercules, CA, USA) as previously described [25]. For first-strand complementary DNA (cDNA) synthesis from RNA templates (2.5 μg), the oligo-d(T)_15_ primer (0.5 ng) was used. After heating at 72 °C for 5 min, the solutions were cooled on ice. Following the protocol of Sileks M (Moscow, Russia), reverse transcription was accomplished in a 50 µL reaction mixture containing 200 U M-MLV reverse transcriptase, 1× M-MLV buffer, and 0.24 mM dNTPs. The reaction proceeded at 36 °C for 1 h, followed by 72 °C for 10 min. The cDNA samples were diluted with nuclease-free water at a 1:10 (*v*/*v*) ratio. In addition, each RNA sample was tested in the absence of the M-MLV enzyme as a negative (RNA-RT) control.

### 2.7. Real-Time RT-PCR

A CFX96 (Bio-Rad Laboratories Inc., Hercules, CA, USA) was used with a 2.5 × SYBR green PCR master mix containing ROX as a passive reference dye (Syntol, Moscow, Russia) for quantitative real-time PCR (qPCR) analysis using the gene-specific primer pairs. The gene-specific primer pairs used in qPCR analysis of ROS-related genes were previously reported [23]. The total 25 µL reaction mixture contained 300 nM primer, 1 µL diluted cDNA, and 2.5 mM MgCl_2_ in a 96-well reaction plate. For each PCR reaction, the protocol included the following steps: 3 min at 95 °C, followed by 35 cycles of 10 s at 95 °C, and 30 s at 60 °C. Data were analyzed using CFX Manager Software (Version 1.5; Bio-Rad Laboratories Inc., Hercules, CA, USA). *EF-1α* (AT5G60390) and *GAPDH* (AT1G13440) genes showed the most stable expression patterns in all tested experimental sets and were used as internal controls in the relative comparison analysis of the studied genes. To confirm the absence of contamination, RNA-RT and no-template controls were included. Each run was accompanied by melting curve analysis to verify the absence of primer-dimer artifacts or non-specific products. Analysis was performed using two separate experiments (performed in January and March 2022) with three technical replicates. 

### 2.8. Statistical Analyses

Statistical analysis was performed using Statistica 10.0 (StatSoft Inc., Tulsa, OK, USA), with a statistical significance level of *p* < 0.05. Two independent categories were compared using the Student’s *t*-test, while comparisons among multiple groups were performed using analysis of variance (ANOVA), followed by a multiple comparison protocol. The intergroup comparison was made using Fisher’s protected least significant difference (PLSD) post hoc test.

## 3. Results

### 3.1. WT and hos1^Cas9^ Cells Have Equal ROS Levels in the Dark

To test whether the mutation of the *HOS1* gene could cause changes in the ROS level without exposure to light, WT and *hos1*^Cas9^ plants were cultivated in the dark for one day. ROS levels were then measured using confocal microscopy with H_2_DCF-DA. For confocal microscopy analysis, H_2_DCFDA was selected as a fluorescent dye because it displays the highest signal-to-noise ratio among other commonly used dyes, penetrates cell layers within the leaf, and reacts with several forms of ROS, such as hydrogen peroxide, hydroxyl radicals, superoxide, and peroxynitrite, ensuring the detection of different types of ROS [26]. The epidermal cells from the abaxial side of leaves were analyzed by confocal microscopy as described in Materials and Methods. WT and mutant plants cultivated in the dark showed the same DCF fluorescence intensity (DFI) at 48.0 ± 2.8 and 49.0 ± 2.7, respectively. DCF fluorescence intensity indicates the total ROS level measured as the sum of hydrogen peroxide, hydroxyl radicals, superoxide, and peroxynitrite. To confirm this result, we obtained callus cultures from WT and *hos1*^Cas9^ plants as described in Materials and Methods. Callus cultures were constantly grown in the dark. The calli were cultivated in liquid medium to obtain single cells and small cell aggregates, and steady-state ROS levels in WT and *hos1*^Cas9^ single living cells were monitored using confocal microscopy. This analysis showed that in the absence of light, WT and mutant cells contained equal amounts of ROS, 84 ± 5 and 83 ± 4 DFI in WT and *hos1*^Cas9^ cells, respectively.

### 3.2. WT and hos1^Cas9^ Plants Have Similar Patterns of ROS Accumulation Inside Cells 

**Figure 1:**
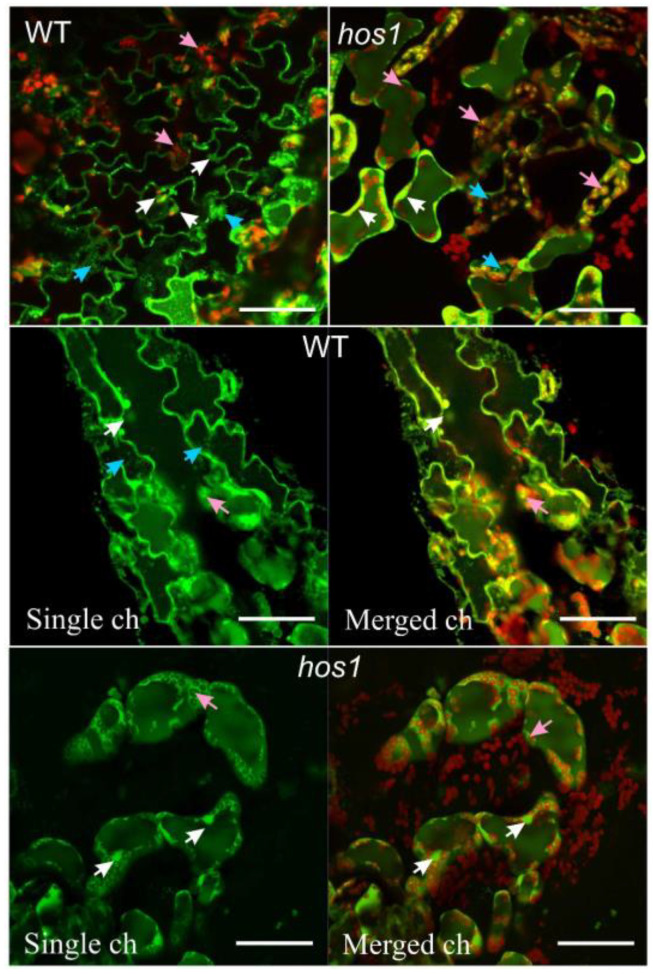
A representative view of ROS localization in epidermal cells of WT and *hos1*^Cas9^ plants. The upper panel shows cell images using two merged channels: the “ROS channel,” in which DCF fluorescence was measured at an excitation wavelength of 488 nm and detected at 505–530 nm, in combination with the “chloroplast channel.” The chloroplast channel represents chloroplast autofluorescence, which was recorded using an emission channel at 600 nm. Chloroplasts are visible as red organelles with green inclusions reflecting ROS localization. White arrows indicate ROS localization in the nucleus (green color), pink arrows indicate chloroplasts (red color), and blue arrows indicate vesicles with ROS (green color). Two bottom panels show images of WT and *hos1*^Cas9^ epidermal cells in a single ROS detection channel (left) and the same cells in merged ROS and chloroplast channels (on the right). The scale bars are 50 µm.

Confocal microscopy was used to study the distribution of ROS in the leaves of normal and mutant *Arabidopsis* plants. In both WT and *hos1*^Cas9^ plants growing in control conditions, ROS accumulated predominantly in the cytoplasm along the plasma membrane and in the adjacent areas of the apoplast (Figure 1). In addition, ROS were found in nuclei and vesicles. ROS accumulation was detected in the chloroplast stroma and along chloroplast membranes, as shown in the bottom panel of Figure 1, where ROS and chloroplast luminescence channels were combined. No peculiarities were found in the intracellular distribution of ROS in mutant plants, which indicates that WT and *hos1*^Cas9^ plants showed similar patterns of ROS accumulation inside cells.

### 3.3. ROS Levels in WT Plants

**Figure 2:**
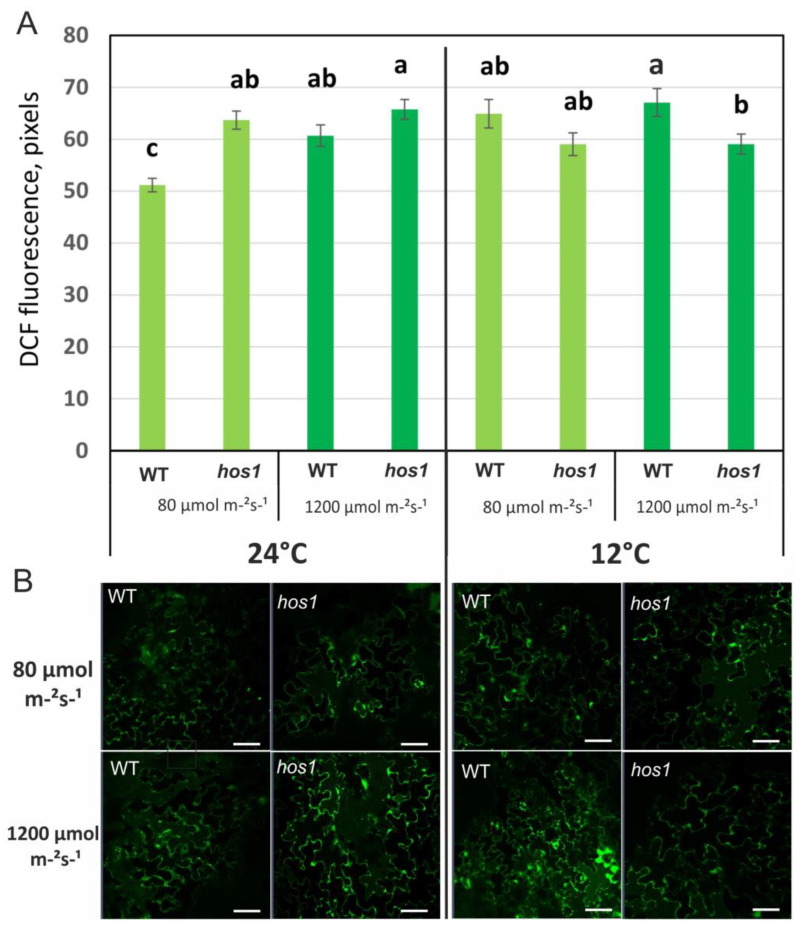
ROS content in epidermal cells from the abaxial leaf side of wild-type (WT) plants, *hos1*^Cas9^ lines of *A*. *thaliana* plants. The plants were loaded with H_2_DCF-DA, and the fluorescence of DCF was visualized by laser-scanning confocal microscopy under control conditions (24 °C/80 µmol m^−2^ s^−1^), high light (24 °C/1200 µmol m^−2^ s^−1^ for 2 h), cold conditions (12 °C for 24 h/80 µmol m^−2^ s^−1^), and high light conditions after cold pre-treatment (12 °C for 24 h, followed by 1200 µmol m^−2^ s^−1^ for 2 h). (**A**) ROS levels are presented as the mean ± SE from three independent experiments. Different letters above the bars indicate significantly different means (*p* < 0.05; Fisher’s LSD). (**B**) A representative view of epidermal cells of wild-type (WT) plants and *hos1*^Cas9^ *A*. *thaliana* plants loaded with H_2_DCF-DA. The brightness of the green fluorescence reflects intracellular ROS abundance. The scale bars are 50 µm.

*Arabidopsis* WT plants were grown in control conditions for 28 days (low light (LL) at 80 µmol m^−2^ s^−1^ and normal growth temperature 24 °C), and then analyzed by confocal microscopy. The program LSM 510 Release 3.5 in the counting algorithm takes into account the size of the cells, the ratio of the cytoplasm and vacuoles, the threshold, and calculates the average value and standard error for individual cells but not for the area of the entire image (see Section 2). Confocal laser imaging of intracellular ROS showed that the ROS level in cells of WT plants increased under conditions of HL (Figure 2A). This result was confirmed in additional experiments with dihydrorhodamine 123 (H2R123) as a probe. Rhodamine-based fluorescent probes are mostly sensitive to H_2_O_2_ and often used in monitoring intracellular ROS [27]. We found that the content of ROS is increased in wild-type plants under intense illumination (Supplementary Figure S3). In conditions of cold or cold treatment followed by HL, a statistically significant increase in ROS level by 20% was observed in WT plants (Figure 2A). 

### 3.4. ROS Levels in hos1^Cas9^ Plants

*Arabidopsis hos1*^Cas9^ plants were grown for 28 days in control conditions (24 °C/80 µmol m^−2^ s^−1^) and then analyzed by confocal microscopy. In *hos1*^Cas9^ plants, ROS levels were increased in control conditions by 22–27% compared to WT plants (Figure 2A, Supplementary Figure S3). Under HL treatment, the ROS content in WT and mutant plants was similar (Figure 2A, Supplementary Figure S3). This means that under LL, the mutant plants increased the level of ROS, but when exposed to strong light, they could no longer increase the level of ROS. Under conditions of cold exposure, an opposite trend was observed, such as a decrease in the level of ROS in mutant plants compared to WT plants. The ROS level in *hos1*^Cas9^ plants under cold treatment followed by HL was 86% of that in WT plants. Measurements carried out on other mutant plants, line *hos1-3* from the SALK collection [10], showed similar trends (Supplementary Figure S4). These results show that the *hos1* mutation increases ROS level, but cold treatment attenuates this effect.

### 3.5. Dynamics of ROS Accumulation under High Light Conditions

#### 3.5.1. The *hos1* Mutation Has a Biphasic Effect on the Level of ROS

**Figure 3:**
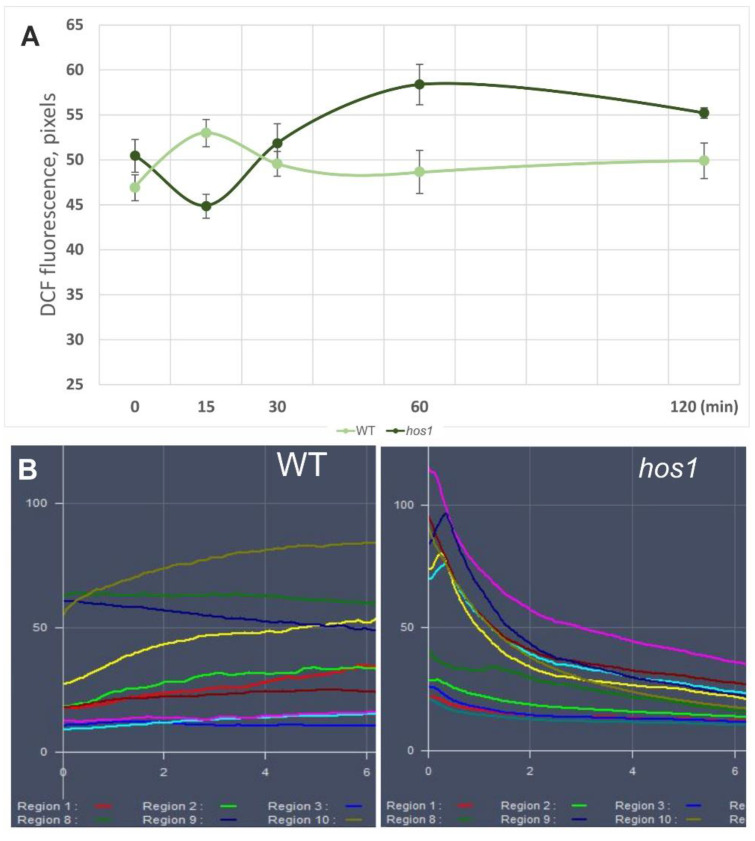
The *hos1*^Cas9^ mutation has a biphasic effect on the level of ROS under HL illumination. Before these experiments, plants were grown at control conditions (24 °C/80 µmol m^−2^ s^−1^). (**A**) ROS dynamics during the 2-h incubation of *Arabidopsis* plants at intense light 1200 µmol m^−2^ s^−1^. A statistically significant difference from the mean of two replicates was observed at 15-, 60-, and 120-min intervals (Student’s *t*-test, *p* < 0.05). (**B**) Different dynamics of ROS accumulation in WT and *hos1*^Cas9^ plants epidermal cells under high-intensity argon laser illumination. X-axis shows DCF fluorescence; Y-axis shows time in minutes. Each color line represents DCF fluorescence in the region of interest (ROI) of an individual cell. Approximately half of the *hos1*^Cas9^-mutant cells initially show high levels of ROS, but then the ROS content steadily decreases. Full 15-min scans and ROI of the analyzed cells are presented in Supplemental Figure S5.

Previous experiments have shown that the *hos1*^Cas9^ mutation can alter ROS levels regardless of cold exposure (Figure 2). Next, we measured ROS levels under HL over a time series ranging from 15 min to 2 h. Under intense illumination of WT plants, the ROS level rapidly increased up to 15 min, and then stabilized at a constant level (Figure 3A). On the contrary, in *hos1* plants, a decrease in the level of ROS was observed in the first 15 min and then increased, reaching a plateau by the 60th min (Figure 3A).

To study the first phase of ROS decline, we examined ROS levels in individual cells, using 2-min intervals (Figure 3B). Epidermal cells of WT and *hos1*^Cas9^ plants were stressed using the Ar laser (488 nm) with increased intensity (see Materials and Methods). The laser intensity was chosen to cause an increase in ROS but not cell damage. ROS accumulation was initially evident at the periphery of the cells and subsequently spread throughout, excluding the vacuoles. A gradual rise in ROS levels was observed for most studied WT cells during the first 10 min, after which the level of ROS stabilized (Supplementary Figure S5). Cells of *hos1*^Cas9^ plants demonstrated completely different trends (Figure 3B). A rapid ROS accumulation was observed in some cells of *hos1*^Cas9^ plants for 30 s before the ROS content dropped significantly. 

Both of these experiments show a biphasic effect of *hos1* mutation on ROS levels, i.e., an initial drop and then an increase in ROS levels. We interpreted these data in the sense that *HOS1* is required to maintain steady-state ROS levels under strong illumination.

#### 3.5.2. The *hos1*^Cas9^ Mutation Prevents the Rise in ROS Levels Caused by Paraquat

Since the decrease in ROS in mutant plants below the norm in the first 15 min of HL illumination could be due to the fact that the mutant plants were better prepared for HL treatment (for example, by the presence of a pre-existing pool of antioxidants), we treated WT and mutant plants with paraquat to cause ROS induction. The herbicide paraquat acts as a terminal oxidant of photosystem I. In light, it reduces oxygen to the superoxide radical, which subsequently dismutates to hydrogen peroxide, inducing stable ROS elevation in treated plant tissues [28]. In *Arabidopsis* plants, a 25 μM paraquat treatment induced rapid and severe oxidative stress [29].

**Figure 4:**
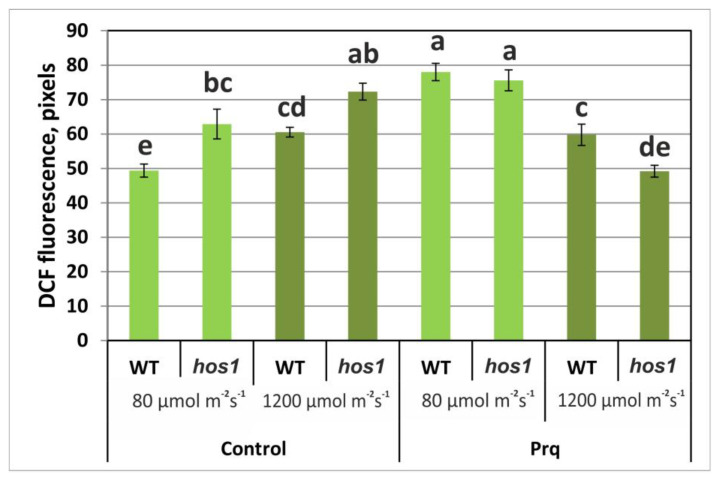
ROS levels in epidermal cells from the abaxial leaf side of WT and *hos1*^Cas9^ lines of *A*. *thaliana* plants treated with 10 μM paraquat under low and high light conditions. Data were obtained using confocal microscopy. The plants were loaded with H_2_DCF-DA, and the fluorescence of DCF was visualized by laser-scanning confocal microscopy under control conditions (24 °C/80 µmol m^−2^ s^−1^) and high light (24 °C/1200 µmol m^−2^ s^−1^ for 2 h). ROS levels are presented as the mean ± SE from three independent experiments. Different letters above the bars indicate significantly different means (*p* < 0.05; Fisher’s LSD).

The experimental setup used in our experiment included moderate treatment conditions (15 μM paraquat and 1 h of light incubation), in which paraquat caused a 1.5-fold increase in ROS in WT plants but did not lead to cell death. As shown in Figure 4, paraquat caused an increase in the ROS content in the cells of WT and *hos1*^Cas9^ plants under LL. HL attenuated paraquat-induced ROS generation in both plant lines. However, this effect on the mutant line was more pronounced. This indicates that the mutant plants generated a stronger antioxidant defense response when treated with HL compared to the wild-type plants. Since paraquat treatment leads to the weakening of chloroplast defense mechanisms, including the rapid oxidation of ascorbate and glutathione [30], the antioxidant status was measured.

#### 3.5.3. Antioxidant Status

ROS is mitigated by an array of ROS-scavenging enzymes and pathways, such as Fe- and CuZn-SODs and the glutathione–ascorbate cycle, as well as high concentrations of antioxidants such as ascorbic acid and reduced glutathione (GSH) [31]. The reduction of H_2_O_2_ via the ascorbate–glutathione pathway is thought to be the major mechanism leading to oxidized glutathione (GSSG) accumulation in plants [31,32]. The balance between GSH and GSSG is a central factor in maintaining the cellular redox state [33]. When the intensity of a stress increases, GSH concentrations decline, and the redox state becomes more oxidized, leading to the deterioration of the system. Measurements of antioxidants were carried out by LC-UV-MS/MS as described in Materials and Methods (see also Supplementary Figure S6).

Measurements of GSH and GSSG, and calculation of their ratios showed that under 15-min HL treatment, both WT and *hos1*^Cas9^ plants significantly decreased GSH levels and increased GSSG levels (Table 1). This result is in good agreement with the data of Haber et al. [32] indicating that the chloroplastic glutathione redox potential was rapidly decreased (within minutes) when *Arabidopsis* plants were shifted from 120 µmol m^−2^ s^−1^ to high light intensity. Glutathione oxidation resulted in a decrease in the GSH/GSSG ratio from 12 to 2.1 for WT plants and from 17 to 3.8 for *hos1*^Cas9^ plants. However, under 2-h HL illumination, both WT and mutant plants partially restored the reduced glutathione pool and returned to the initial GSH/GSSG ratios. According to the literature data, *Arabidopsis* leaves in normal physiological conditions contain 152–263 nmol g^−1^ FW of GSH and 21–75 nmol g^−1^ FW of GSSG, thus maintaining the GSH/GSSG ratio in the range of 2.0–12.5 [34]. Our data imply that when exposed to strong light, *Arabidopsis* plants rapidly deplete the pool of reduced glutathione at first but then adapt to light stress. The difference in the content of GSH in normal and mutant plants is significant and lies in the fact that mutant plants contain 1.3 times less GSH. However, since the dynamics of depletion and recovery are similar between WT and *hos1*^Cas9^ plants, we concluded that the *hos1* gene mutation does not change the redox balance in this parameter.

*Arabidopsis* plants (ecotype Col-0) normally contain ascorbic acid (AsA) at a level between 2 and 3 µmol g^−1^ FW (2000 and 3000 nmol g^−1^ FW; [35,36]). The HPLC/MS measurements of AsA levels showed 2087 nmol g^−1^ FW of AsA in WT plants (Table 1). In *hos1*^Cas9^ plants, the level of AcA was twice as high and amounted to 4213 nmol g^−1^ FW. This may be a compensation for reduced GSH levels in *hos1*^Cas9^ plants, resembling an effect previously observed by Müller-Moulé et al. [37], when the lack of one reducing agent is compensated by another. A decrease in the concentration of AsA in mutant plants should be noted at a 15-min HL exposure. Under two hours of HL irradiation, both plant lines accumulated more AsA, reaching 5539 and 5534 nmol g^−1^ FW of AsA in WT plants and *hos1*^Cas9^ plants, respectively. This corresponds to the literature data, which indicate that under strong light, the content of AsA in *Arabidopsis* increases [37,38]. Thus, the *hos1* mutation significantly increases AsA content but generally disrupts normal GSH and AsA levels in both control and HL conditions, indicating that HOS1 is required to maintain normal redox homeostasis.

### 3.6. NADPH Oxidase Gene Expression

**Figure 5:**
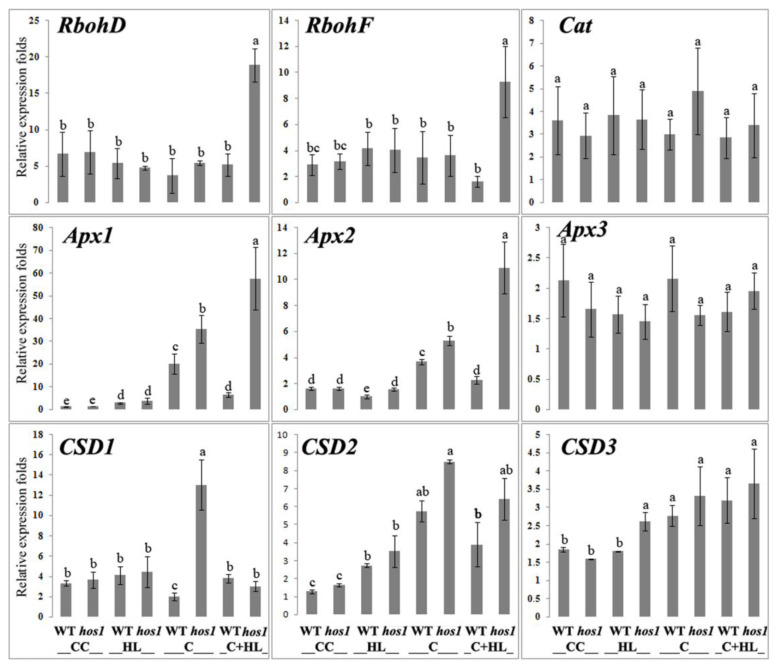
Expression of *Rboh* genes and genes encoding ROS-detoxifying enzymes in WT and *hos1*^Cas9^ plants. The genes encoding ROS-detoxifying enzymes are presented by superoxide dismutase (SOD, *CSD1-3*), ascorbate peroxidase (*APX1-3*), and catalase (*Cat1*). Data are presented as the mean ± SE from the analysis of two different experiments with three technical replicates. Different letters above the bars indicate significantly different means (*p* < 0.05; Fisher’s LSD). **CC**, control conditions (24 °C/80 µmol m^−2^ s^−1^); **HL**, high light (24 °C/1200 µmol m^−2^ s^−1^ for 2 h); **C**, cold conditions (12 °C for 24 h/80 µmol m^−2^ s^−1^); **C + HL**, cold and high light conditions (12 °C for 24 h, followed by 1200 µmol m^−2^ s^−1^ for 2 h).

ROS are generated in a response to stress by NADPH oxidases (Rboh) at the plasma membrane of *Arabidopsis* plants, and enhanced photoprotection occurs due to the induction of an apoplastic H_2_O_2_ burst by activating NADPH oxidase expression [39]. Two NADPH oxidases, RbohD and RbohF, play the most important role in light acclimation [21,40]. The different systemic responses observed during HL-induced acclimation could be divided into RBOHD-dependent and RBOHD-independent responses [41]. The hypothesis that the *hos1* mutation affects the expression of genes encoding NADPH oxidases was tested by qPCR. The analysis showed that, in control conditions (24 °C/80 µmol m^−2^ s^−1^), the expression of *RbohD* and *RbohF* was similar in WT and *hos1*^Cas9^ *Arabidopsis* plants (Figure 5). Similarly, light or cold acting separately did not significantly affect *RbohD* and *RbohF* expression. However, when plants were pre-exposed to cold and further exposed to HL, the expression of *RbohD* and *RbohF* was significantly increased in *hos1*^Cas9^ plants but not in WT plants.

### 3.7. Intense Light Has Little Effect on the Expression of Genes Involved in ROS Detoxification

Because ascorbate peroxidase (APX) relies on electrons from reduced glutathione, channeled to APX through the ascorbate–glutathione cycle [31], we measured the expression level of genes encoding APX and other ROS-detoxifying enzymes. The detection of sustained, moderately elevated ROS levels usually means that the expression of ROS-generating enzymes is compensated by the antioxidant system [41,42]. Enzymatic ROS-scavenging mechanisms include the induction of superoxide dismutase (SOD), ascorbate peroxidase (APX), and catalase (CAT) [43]. Using qPCR, we tested whether marker genes, such as *Apx1*, *Apx2, Apx3*, *CSD1*, *CSD2*, *CSD3*, and *CAT1,* were activated or inhibited in *hos1*^Cas9^ plants. qPCR measurements showed almost complete insensitivity of these marker genes to the *hos1* mutation when plants were cultivated under normal temperature conditions (Figure 5). At the same time, an increase in illumination (HL conditions) slightly stimulated the expression of *Apx2* and *CSD3* in *hos1*^Cas9^ plants compared to WT plants (Figure 5).

### 3.8. Cold Significantly Activates Expression of Genes Encoding Ascorbate Peroxidases 

When WT and *hos1*^Cas9^ plants were cultivated in the cold, the expression of *Apx1* and *Apx2* increased significantly (Figure 5). This was especially noticeable for *Apx1*. The level of *Apx1* transcripts increased in the cold 10-fold and 16-fold in WT and mutant plants, respectively, compared with normal cultivation conditions. Only *Apx3* did not change the level of expression under the influence of cold. The expression of *CSD1* increased sixfold in *hos1*^Cas9^ versus WT plants. The hos1 mutation had no effect on CSD2 or CSD3 expression (Figure 5). 

A 24-h cold treatment followed by intense illumination led to a state in which the expression of *Apx1* and *Apx2* genes in WT plants was almost at the basal level under normal conditions. The *hos1* mutation eliminated this effect, causing a significant increase in the expression of *Apx1* and *Apx2*. In mutant plants, *Apx1* and *Apx2* appear to be induced as a response to increased ROS levels produced via enhanced *RbohD* and *RbohF* expression. In contrast to the *Apx1* and *Apx2* genes, *CSD* genes were not significantly affected by the *hos1* mutation under cold with HL treatment. Based on these findings, we concluded that the *hos1* mutation has an activatory effect on the expression of *CSD1* under cold conditions and *Apx1* and *Apx2* under cold conditions, followed by HL.

## 4. Discussion 

The basal level of ROS is necessary for normal plant development and maintaining the basal level of ROS below the cytotoxic level but above the cytostatic level is an important biological process [44,45]. In this work, we studied the effect of the *hos1* mutation on ROS content in *A. thaliana* WT and *hos1*^Cas9^ mutant plants. Our goal was to attenuate the functioning of the pleiotropic regulator HOS1, which is required for adaptation of plant development to environmental temperature [1,2,3,4,5,6,7], and to see against this background if *HOS1* manifests its effects through ROS modulation.

The *hos1*^Cas9^ mutant plants showed complex dynamics of ROS accumulation depending on external stimuli. In the absence of light and at normal growth temperatures, the level of ROS does not change in mutant plants relative to WT plants. The level of ROS was affected differently by cold or light. LL and HL caused an increase in ROS in *hos1*^Cas9^ plants, similarly affecting ROS production (Figure 2), while cold caused processes similar to acclimation, which led to the stabilization of ROS at a lower level compared with WT plants (Figure 2).

**Figure 6:**
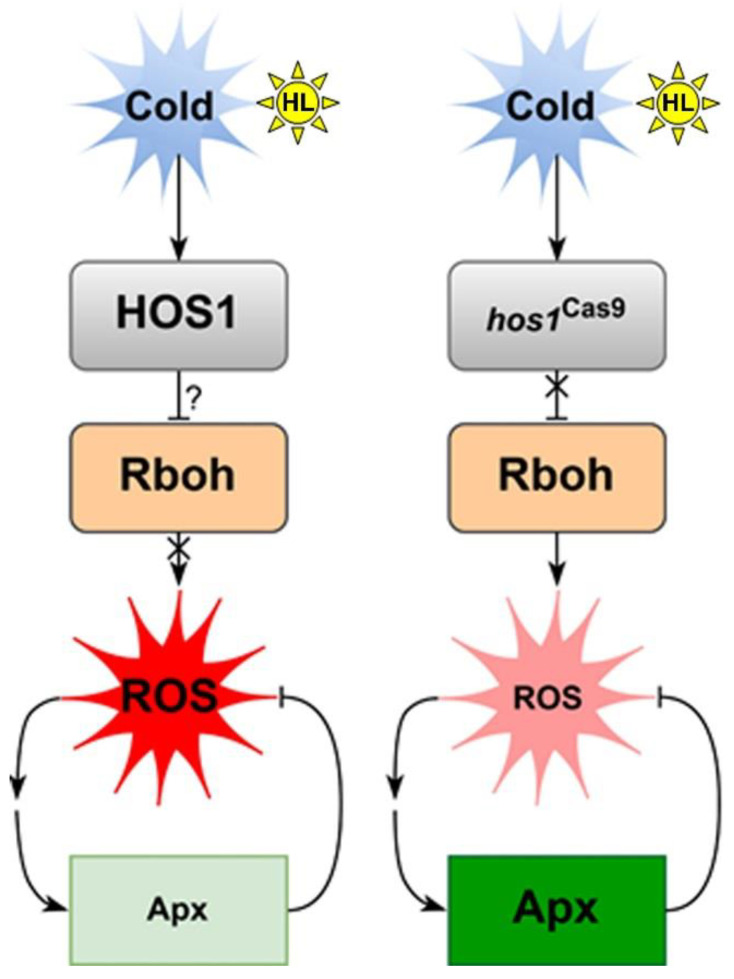
A working model showing putative signal transduction mediated by the HOS1 protein under cold exposure and strong lighting. It can be supposed (?) that when exposed to cold and strong light, HOS1 stabilizes the content of ROS by blocking its generation through NADPH oxidases (*RbohD/F*). As a result, there is no increased generation of ROS and no activation of ROS-regulated expression of ascorbate peroxidases *Apx1* and *Apx2*. Inactivation of HOS1 removes (X) this regulatory block. The expression of *RbohD/F* activates the pathway of ROS-regulated expression of *Apx1* and *Apx2*, which leads to a decrease in the content of ROS by active ROS decomposition.

The cold-dependent way of regulating ROS metabolism in *hos1*^Cas9^ plants was most pronounced when plants were exposed to cold and then to HL. Figure 6 shows the putative pattern of HOS1-mediated signaling when *Arabilopsis* plants are exposed to cold and strong light. Under these conditions, WT plants did not maintain the initial ROS level, and the ROS level increased (Figure 2A). However, *hos1*^Cas9^ plants treated with cold followed by HL maintained a low level of ROS (Figure 2A). This effect was accompanied by an increased expression of *RbohD* and *RbohF* and a concomitant increase in the expression of *Apx1* and *Apx2* (Figure 5). Probably, the initial rise of ROS, initiated by increased expression of *RbohD* and *RbohF,* was compensated by ascorbate peroxidases. Several studies [18,39,40] reported that RbohD and RbohF play important roles in cold and light acclimation. Since the maintenance of an optimal level of ROS is necessary for the proper functioning of cells [44], HOS1 can serve as a limiting factor to prevent intense changes in the level of ROS. This can be implemented using the mechanism proposed by Davletova et al. [46], where RbohD is involved in the Apx1 signaling pathway as a positive amplification factor that maintains the expression of *Apx1* transcripts at a high stationary level.

While the effect of the *hos1* mutation on light signaling components and the relationship between photoperiodic flowering and cold in *hos1* mutant plants have been previously shown [10,11,12], the effect of HL on *hos1*-mutated plants was identified for the first time in this study. The adaptation processes of *hos1*^Cas9^ plants to intense light are different from those caused by cold. Increased ROS production is not accompanied by the induction of pro- and antioxidant genes (Figure 5). Rather, *hos1*^Cas9^ plants demonstrate pre-formed antioxidant defense. It is known that light illumination leads to the formation of reduced forms of oxygen, such as superoxide and excited chlorophyll molecules, which generate singlet oxygen. Superoxide can be converted into hydrogen peroxide and hydroxyl radicals. These light-induced ROS are amplified when the absorption of light energy becomes excessive relative to the photosynthetic activity [43,47]. The first line of defense against excessive ROS is the nonenzymatic ROS-scavenging mechanism, which include scavengers such as ascorbate, glutathione, carotenoids, tocopherol, and flavonoids [43].

We suggest that the *hos1* mutation activates the first line of defense against HL. This is supported by several observations: (1) Changes in ROS levels in *hos1*^Cas9^ plants are rapid processes occurring within minutes, including biphasic fluctuations in ROS levels (Figure 3). Such rapid responses could be explained by the depletion of a pre-existing pool of antioxidants. (2) Under HL conditions, paraquat did not increase the accumulation of ROS (Figure 4), which can be explained by the presence of a high pool of antioxidants. (3) *hos1*^Cas9^ plants contain a high content of ascorbic acid (Table 1) and (4) *hos1*^Cas9^ plants contain a high content of flavonoids [9].

It is clear that the influence of the *HOS1* gene on ROS can be associated with various regulatory mechanisms, including both ABA signaling and cues from the light signaling system. The most promising for further study are the regulatory modules HOS1—‖ICE1—‖ABI5→*RbohD*/*F* and HOS1—‖PIF4→*ABI5*→*RbohD*/*F*, which may have an overlap in ROS regulation via ABI5 function [48]. Similarly, the HOS1-RBOHD/F-ROS-ENO2-CBF signaling chain may explain how HOS1 activates plant cold response through ENOLASE2 (ENO2), given the recently discovered role of ENO2 in cold adaptation [49].

## 5. Conclusions

Changes in ROS levels mediated by the *hos1* mutation seem to have complex behavior due to the cross-talk of several levels of regulation. Such levels of regulation could be the influence of the gene mutation itself, the compensatory mechanisms of plants, and the participation of signaling pathways triggered by external stimuli, such as cold or light. It is possible that HOS1 prevents the overinduction of defense mechanisms to balance growth. HOS1 is required to maintain ROS homeostasis not only under cold conditions, but also under conditions of both low and high light intensity. It is likely that *HOS1* affects ROS metabolism through different mechanisms under cold or HL conditions.

## Figures and Tables

**Table 1:** Content of reduced and oxidized glutathione (GSH and GSSG) and ascorbic acid in WT and *hos1*^Cas9^ plants in control conditions (24 °C/80 µmol m^−2^ s^−1^) and HL conditions (24 °C/1200 µmol m^−2^ s^−1^). The data are presented as nmol/g fresh weight ± SE from two separate experiments.

	Control Conditions		HL, 15 Min		HL, 120 Min	
	WT	*hos1* ^Cas9^	WT	*hos1* ^Cas9^	WT	*hos1* ^Cas9^
Ascorbic acid	2087 ± 312	4213 ± 47	5210 ± 60	3108 ± 249	5539 ± 85	5374 ± 166
GSH	258 ± 28	199 ± 16	121 ± 17	125 ± 15	157 ± 8	161 ± 14
GSSG	20.9 ± 2.9	11.6 ± 1.5	56.6 ± 7.2	32.8 ± 2.2	10.5 ± 0.8	13.1 ± 1.6

## Data Availability

The datasets generated during and/or analysed during the current study are available from the corresponding author on reasonable request.

## Supplementary Materials

The following supporting information can be downloaded at: https://www.mdpi.com/article/10.3390/life13020524/s1, Reference [50] are cited in the supplementary materials. Table S1: Experimental design; Figure S1: Spectral characteristics of the light-emitting diode lamps used; Figure S2: Suppression of HOS1 in *hos1*^Cas9^ *A. thaliana* plants; Figure S3: ROS content in epidermal cells from the abaxial leaf side of wild-type (WT), and *hos1*^Cas9^ lines of *A. thaliana* plants measured with dihydrorhodamine 123; Figure S4: ROS content in epidermal cells from the abaxial leaf side of wild-type (WT), and *hos1-3* lines mutant from the SALK collection of *A*. *thaliana* plants; Figure S5: Dynamics of ROS accumulation in epidermal cells of WT and *hos1*^Cas9^ plants under high-intensity argon laser illumination; Figure S6: HPLC chromatograms of ascorbic acid (ASA), reduced and oxidized glutathione (GSH and GSSG) in *Arabidopsis* WT and *hos1*^Cas9^ plants at the control condition.
