# Supplementary material for: Suppression of the HOS1 Gene Affects the Level of ROS Depending on Light and Cold
Source: Life (Basel). 2023 Feb 14;13(2):524. doi: 10.3390/life13020524 (PMC9960889; doi:10.3390/life13020524)
Supplement: Supplementary file 1 [file life-13-00524-s001.zip › life-2120433-supplementary.pdf]

Supplementary Information

Suppression of the *HOS1* gene affects the level of ROS depending on light and cold

T.Y. Gorpenchenko[1], G.N. Veremeichik[1*], Y.N. Shkryl[1], Y.A. Yugay[1], V.P. Grigorchuk[1]. D.V. Bulgakov[1], T.V. Rusapetova[1], Y.V. Vereshchagina[1], A.A. Mironova[1], E.P. Subbotin[2], Y.N. Kulchin[2], V.P. Bulgakov[1*]

[1] *Federal Scientific Center of the East Asia Terrestrial Biodiversity, Far Eastern Branch of the Russian Academy of Sciences, 159 Stoletija Str., Vladivostok, 690022, Russia*

[2] *Institute of Automation and Control Processes, Far Eastern Branch of the Russian Academy of Sciences 5 Radio str., Vladivostok, 690041, Russia*

Content

Supplementary Table S1. Experimental design.

Supplementary Figure S1. Spectral characteristics of the light-emitting diode lamps used.

Supplementary Figure S2. Suppression of HOS1 in *hos1*^{Cas9} *A. thaliana* plants.

Supplementary Figure S3. ROS content in epidermal cells from the abaxial leaf side of wild-type (WT), and *hos1*^{Cas9} lines of *A. thaliana* plants measured with dihydrorhodamine 123.

Supplementary Figure S4. ROS content in epidermal cells from the abaxial leaf side of wild-type (WT), and *hos1-3* lines mutant from the SALK collection of *A. thaliana* plants.

Supplementary Figure S5. Dynamics of ROS accumulation in epidermal cells of WT and *hos1*^{Cas9} plants under high-intensity argon laser illumination.

Supplementary Figure S6. HPLC chromatograms of ascorbic acid (ASA), reduced and oxidized glutathione (GSH and GSSG) in *Arabidopsis* WT and *hos1*^{Cas9} plants at the control condition.

Table S1. Experimental design.

Number of experiment	Plant	Conditions
1	WT	Ambient temperature, normal light 24ºC/80 µmol m^{-2} s^{-1}
2	*hos1*^{Cas9}	
3	WT	Ambient temperature, high light 24ºC/1200 µmol m^{-2} s^{-1} (2 h)
4	*hos1*^{Cas9}	
5	WT	Low temperature, normal light 12ºC (24 h)/80 µmol m^{-2} s^{-1}
6	*hos1*^{Cas9}	
7	WT	Low temperature, high light 12ºC (24 h)/1200 µmol m^{-2} s^{-1} (2 h)
8	*hos1*^{Cas9}	

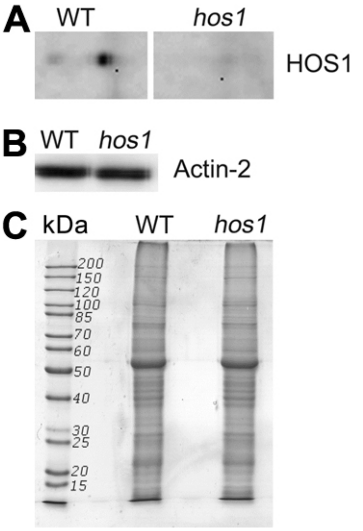

Figure S1. Suppression of HOS1 in *hos1*^{Cas9} *A. thaliana* plants. The levels of HOS1 protein in control and *hos1*^{Cas9} plants are shown in a Western blot: **A**, detection of HOS1 after 2-D immunoblotting in control (WT) and *hos1*^{Cas9} plants (*hos1*). **B**, Actin-2 served as a loading control. **C**, SDS-PAGE stained with Coomassie Brilliant Blue G-250. The intense band of Rubisco (55 kDa) was used as an extra loading control.

Experimental

Antibodies

Anti-HOS1 rabbit polyclonal antibody (PHY0786A, PhytoAB, San Jose, California, USA); Anti-Actin mouse monoclonal antibody (MA1-744, Thermo Fisher Scientific, USA). Goat anti-mouse IgG (H+L) Cross-Adsorbed Secondary Antibody, AP (G-21060, Thermo Fisher Scientific); Goat anti-Rabbit IgG (H+L) Cross-Adsorbed Secondary Antibody, AP (G-21079, Thermo Fisher Scientific).

SDS-PAGE and Western blot

Proteins were isolated from 1 g fresh weight of *A. thaliana* plants using a phenol extraction methanol/ammonium acetate precipitation method as described (Bulgakov et al., 2018). Protein from each extraction type was quantified using Bradford assay.

Prior to electrophoresis, the extracts were diluted in 2× Laemmli's sample buffer. The SDS gel electrophoresis was carried out on 10% gel using the Mini-Protean 3 Cell (Bio-Rad Laboratories, USA). After electrophoresis, proteins were transferred to PVDF membranes (IPVH00010, Merk-Millipore, Germany) by wet blotting using a Mini Trans-Blot Electrophoretic Transfer Cell (Bio-Rad Laboratories) following the manufacturer's instructions. After transfer, a membrane was placed in blocking solution (PBS containing 0.2%[w/v] I-Block™ reagent (T2015, Thermo Fisher Scientific) and 0.1% [v/v] Tween 20) for 1 h at room temperature. The membrane was incubated with the primary antibody diluted in blocking solution for 1 h at room temperature. Immunoreactive bands were detected with the AP-labeled secondary antibody followed by CDP-Star™ Substrate (T2146, Thermo Fisher Scientific) detection reagent.

2-D Gel Electrophoresis

2-D Gel Electrophoresis was performed as described (Bulgakov et al., 2018) [50]. ReadyStrip™ IPG Strips pH 3–10 NL 7cm (1632002, Bio-Rad Laboratories) were used for IEF according to the manufacturer's recommendations.

Bulgakov V.P.; Vereshchagina, Y.V.; Bulgakov, D.V.; Veremeichik, G.N.; Shkryl, Y.N. The *rolB* plant oncogene affects multiple signaling protein modules related to hormone signaling and plant defense. *Sci. Rep.* 2018, 2; *8*, 2285. doi: 10.1038/s41598-018-20694-6.

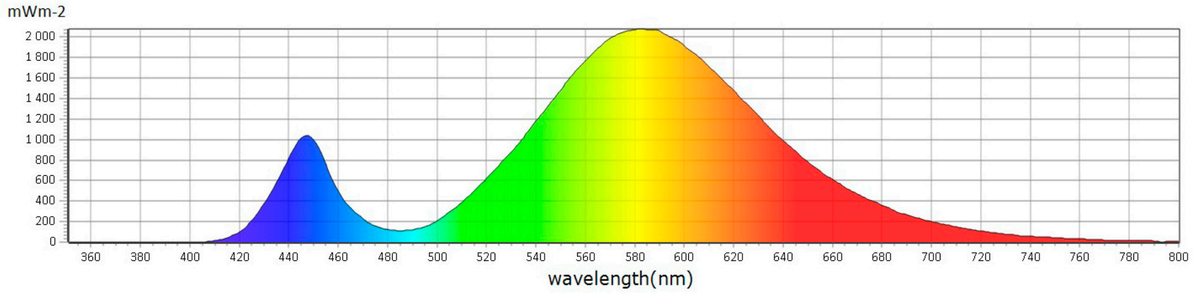

PPFD	: 88,46 umolm-	PPFD	: 1238 umolm-
x	: 0,4535	x	: 0,4338
y	: 0,4213	y	: 0,4146
LambdaD	: 582 nm	LambdaD	: 581 nm
LambdaP	: 598 nm	LambdaP	: 581 nm

Figure S2. Spectral characteristics of the light-emitting diode lamps used. These warm white lamps are characterized by an evenly distributed emission spectrum and have emission maxima at 581 nm and 448 nm.

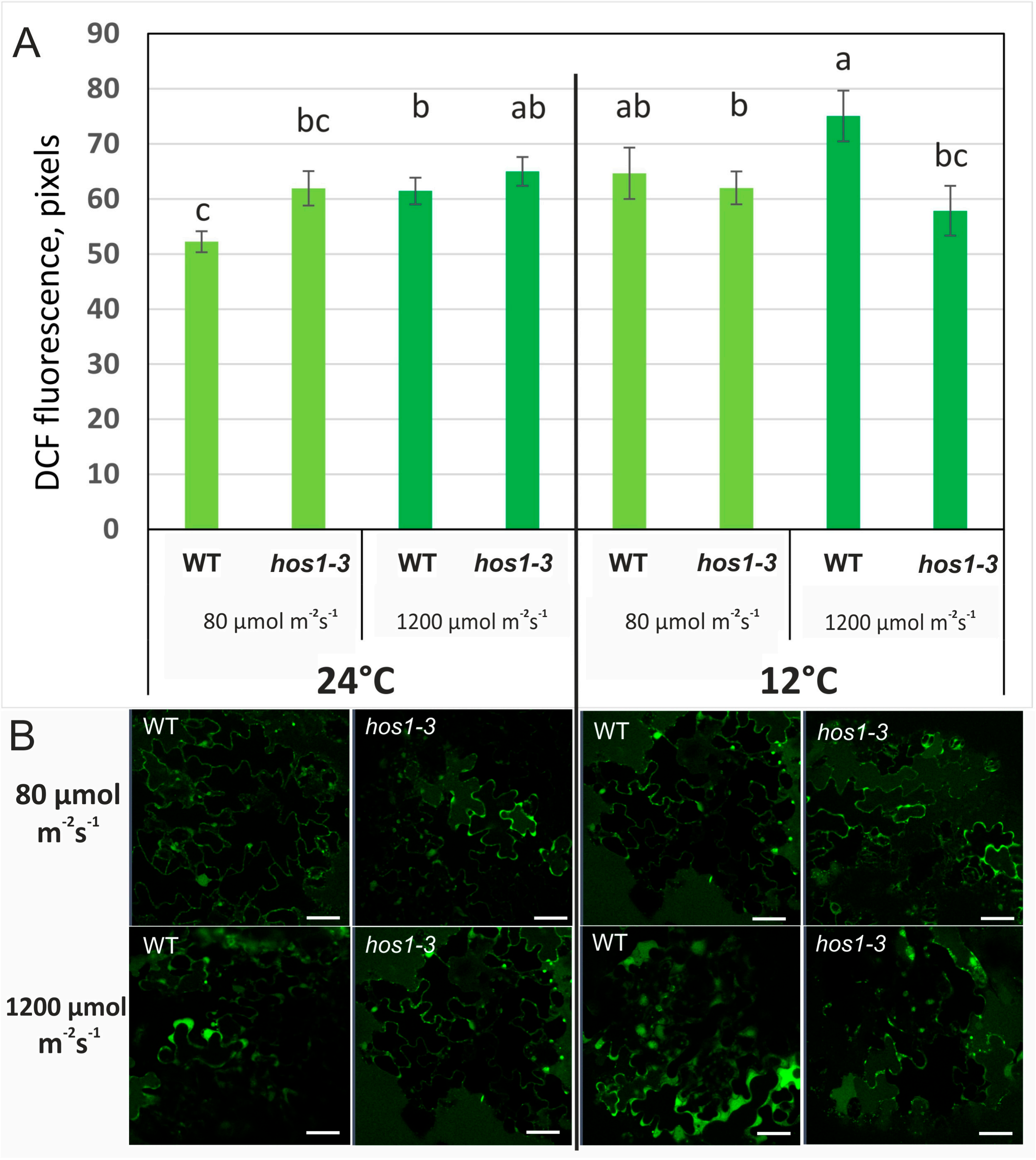

Figure S3. ROS content in epidermal cells from the abaxial leaf side of wild-type (WT), and *hos1*^{Cas9} lines of *A. thaliana* plants. The plants were loaded with dihydrorhodamine 123, and the fluorescence was visualized by laser-scanning confocal microscopy. ROS levels are presented as the mean ± SE from three independent experiments. Different letters above the bars indicate significantly different means ($p < 0.05$; Fisher's LSD). ROS levels were measured under control conditions (24ºC/80 µmol m^{-2} s^{-1}) and high light conditions (24ºC/1200 µmol m^{-2} s^{-1}for 2 h).

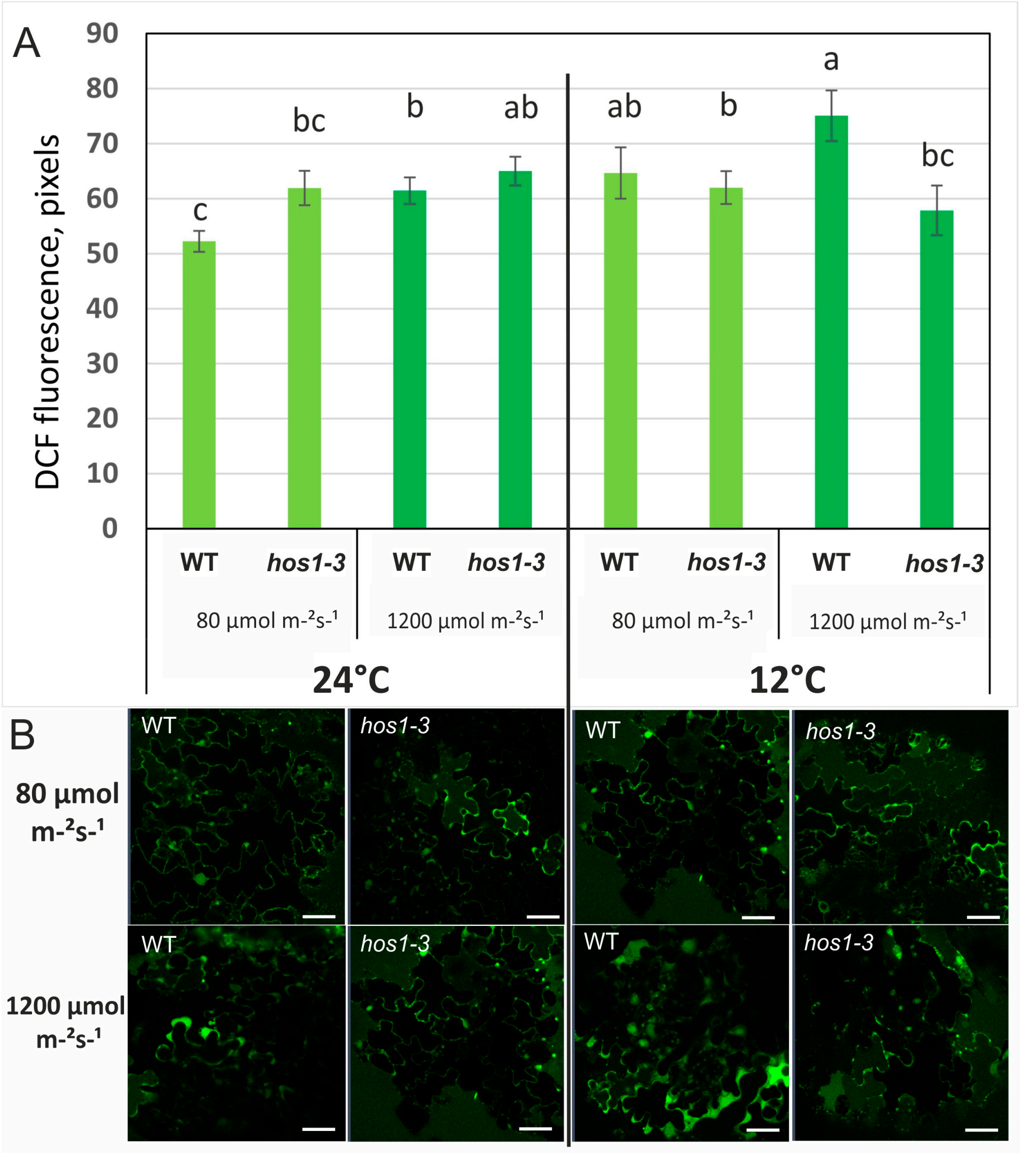

Figure S4. ROS content in epidermal cells from the abaxial leaf side of wild-type (WT), and *hos1-3* line from the SALK collection of *A. thaliana* plants. The plants were loaded with H_2DCF-DA, and the fluorescence of DCF was visualized by laser-scanning confocal microscopy. **A**, ROS levels are presented as the mean ± SE from three independent experiments. Different letters above the bars indicate significantly different means ($p < 0.05$; Fisher's LSD). **B**, representative view of epidermal cells of wild-type (WT) and *hos1-3* mutant line loaded with H_2DCF-DA. The brightness of the green fluorescence reflects intracellular ROS abundance. The scale bars are 50 µm. ROS levels were measured under control conditions (24°C/80 µmol m^{-2} s^{-1}); high light (24°C/1200 µmol m^{-2} $s^{-1}$for 2 h) and after cold treatments (12°C for 24 h/80 µmol m^{-2} s^{-1} and 12°C for 24 h, followed by 1200 µmol m^{-2} s^{-1} for 2 h).

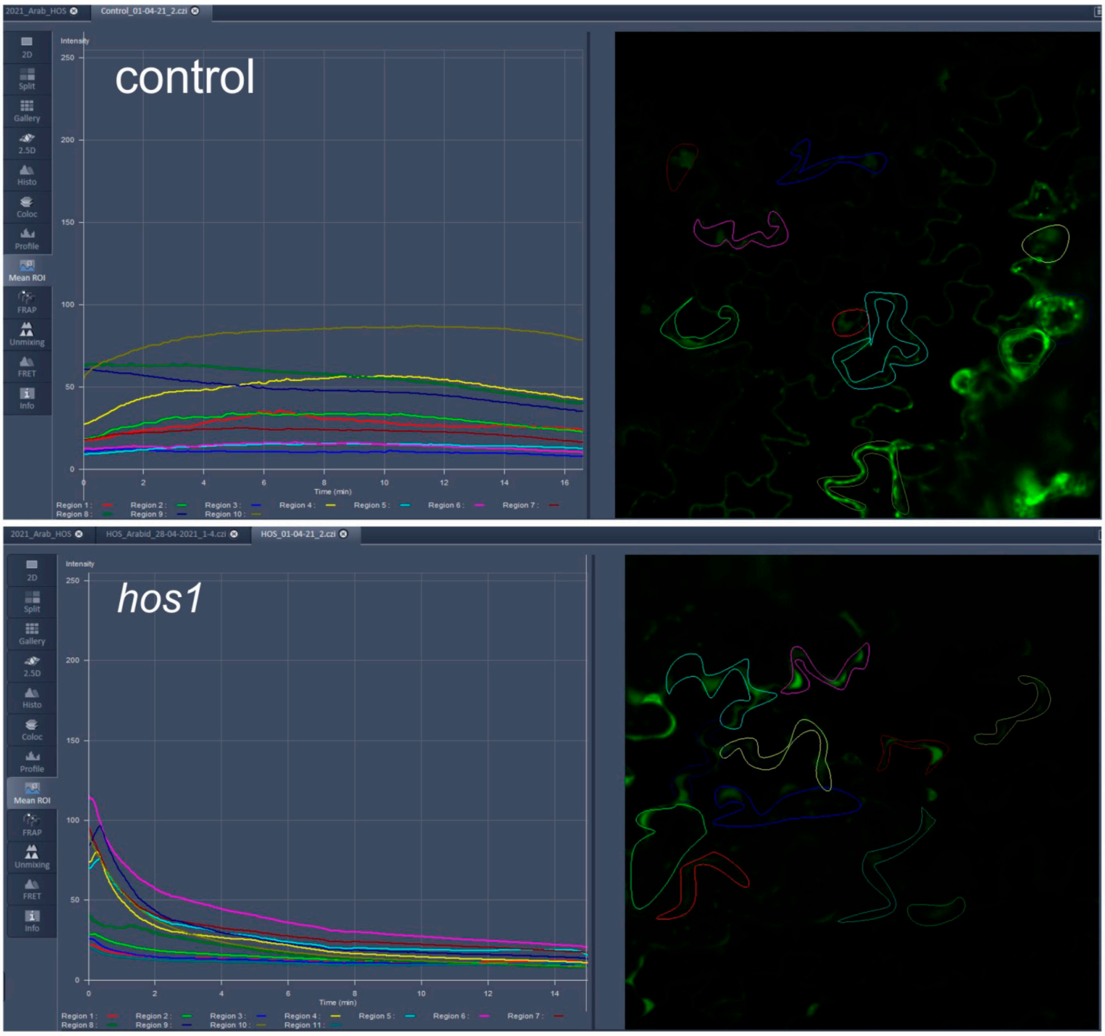

Figure S5. Dynamics of ROS accumulation in epidermal cells of WT and *hos1*^{Cas9} plants under high-intensity argon laser illumination. The results of a 15-minute cell exposure under the influence of a high-intensity argon laser are presented. X-axis shows DCF fluorescence, Y-axis shows time in minutes. Each color line represents DCF fluorescence in the region of interest (ROI) of an individual cell. Before experiment, plants were grown at control conditions (24ºC/80 µmol m^{-2} s^{-1}).

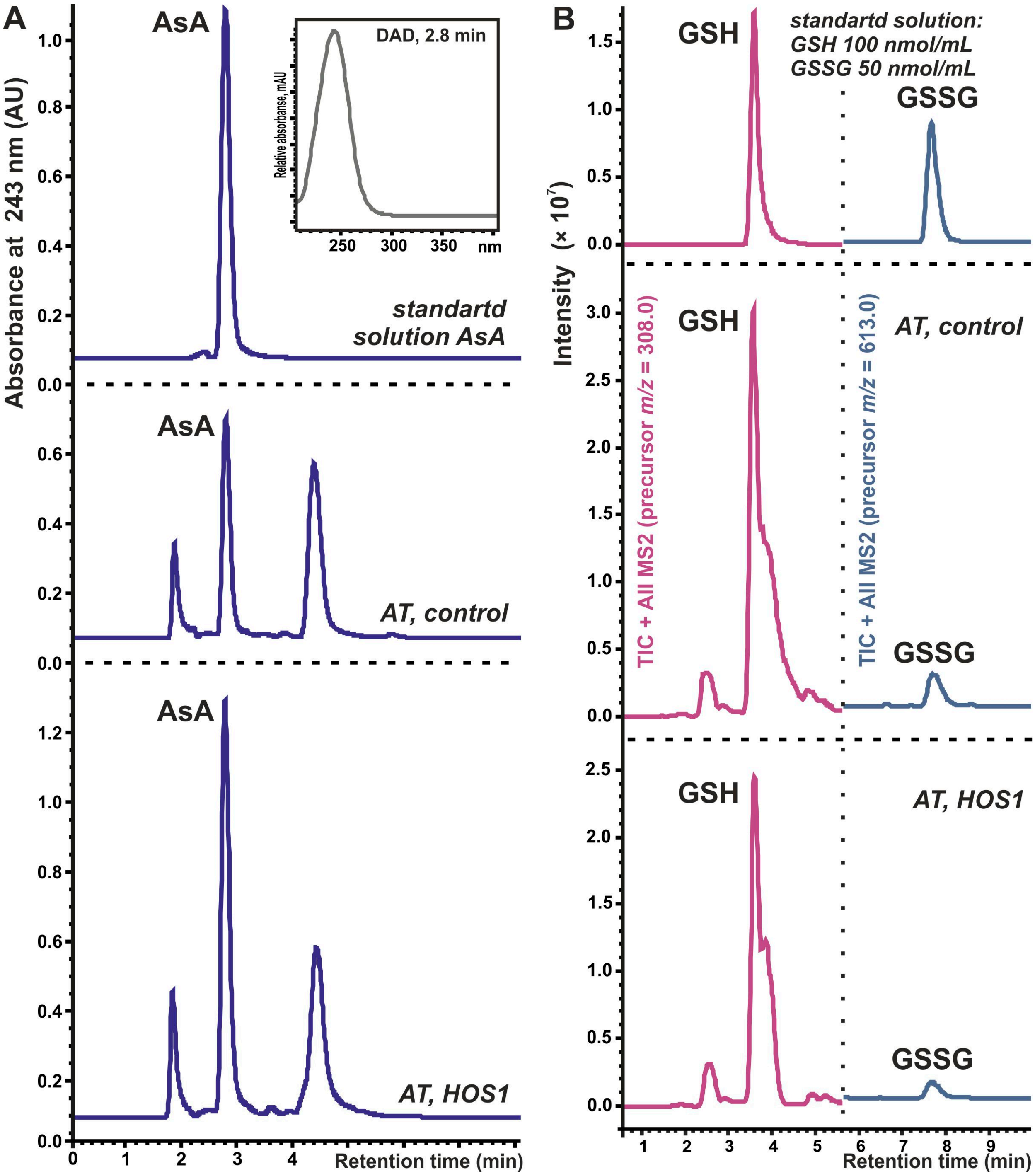

Figure S6. HPLC chromatograms showing profiles of ascorbic acid (ASA), reduced and oxidized glutathione (GSH and GSSG) in *Arabidopsis* WT and *hos1*^{Cas9} plants at the control condition.
